# Fibroblast growth factor 21 as a biomarker for long-term complications in organic acidemias

**DOI:** 10.1007/s10545-018-0244-6

**Published:** 2018-08-29

**Authors:** F. Molema, E. H. Jacobs, W. Onkenhout, G. C. Schoonderwoerd, J. G. Langendonk, Monique Williams

**Affiliations:** 1 Department of Pediatrics Sophia Children’s Hospital, Center of Lysosomal and Metabolic Disorders, Erasmus University Medical Center Rotterdam, Postbus 2060, 3000 CB Rotterdam, The Netherlands; 2 Department of Clinical Genetics, Erasmus University Medical Center, Rotterdam, The Netherlands; 3 Center of Lysosomal and Metabolic Disorders, Department of Internal Medicine, Erasmus University Medical Center, Rotterdam, The Netherlands

## Abstract

**Background:**

There is increasing evidence that long-term complications in organic acidemias are caused by impaired mitochondrial metabolism. Currently, there is no specific biomarker to monitor mitochondrial dysfunction in organic acidemias. Serum fibroblast growth factor 21 (FGF-21) is a biomarker for mitochondrial disease and could be a candidate to monitor mitochondrial function in the deleterious course of disease.

**Methods:**

Data of 17 patients with classical organic acidemias (11 propionic acidemia (PA), four methylmalonic acidemia (MMA) and two isovaleric acidemia (IVA) patients) were included. The clinical course was evaluated; metabolic decompensations and long-term complications were correlated with plasma FGF-21 levels. Cardiomyopathy, prolonged QT interval, renal failure, and optic neuropathy were defined as long-term complications.

**Results:**

Patients ages ranged from 16 months up to 32 years. Serious long-term complications occurred in eight patients (five PA and three MMA patients). In MMA and PA patients plasma FGF-21 levels during stable metabolic periods were significantly higher in patients with long-term complications (*Mdn* = 2556.0 pg/ml) compared to patients without (*Mdn* = 287.0 pg/ml). A median plasma FGF-21 level above 1500 pg/ml during a stable metabolic period, measured before the occurrence of long-term complications, had a positive predictive value of 0.83 and a negative predictive value of 1.00 on long-term complications in MMA and PA patients.

**Conclusion:**

This study demonstrates the potential role of FGF-21 as a biomarker for long-term complications in classical organic acidemias, attributed to mitochondrial dysfunction.

**Electronic supplementary material:**

The online version of this article (10.1007/s10545-018-0244-6) contains supplementary material, which is available to authorized users.

## Introduction

The branched chain amino acids isoleucine, valine, and leucine are essential nutrients for human growth and development (Adibi 1976). The enzymes methylmalonyl-CoA mutase, propionyl-CoA carboxylase, and isovaleryl-CoA dehydrogenase play pivotal roles in the metabolism of these amino acids. Deficiencies of these enzymes result in three classic organic acidemias, methylmalonic acidemia (MMA; OMIM #251000, #251100, #251110, #277400, #277410), propionic acidemia (PA; OMIM #606054), and isovaleric acidemia (IVA; OMIM #243500). MMA and PA are characterized by the potentially lethal accumulation of toxic metabolites, mainly 2-methylcitric acid, malonic acid, and propionyl-CoA (Kölker et al. 2003; Schwab et al. 2006).

The treatment mainly consists of protein reduction as well as avoidance of catabolism, and survival has greatly improved beyond childhood (Enns et al. 2007; Pena et al. 2012). However, the prognosis is still unsatisfactory with a high rate of severe long-term complications, such as renal failure, cardiac arrhythmias, cardiomyopathy, optic nerve atrophy with vision loss, pancreatitis, and mental retardation (Marquard et al. 2011; Sutton et al. 2012; Baumgartner et al. 2014; Fraser and Venditti 2016). The protein restricted diet may also cause complications, such as failure to thrive (van der Meer et al. 1996; Yannicelli et al. 2003). Mitochondrial dysfunction has been established in the pathophysiology of all long-term complications (Schwab et al. 2006; Chandler et al. 2009; de Keyzer et al. 2009; Fragaki et al. 2011; Melo et al. 2011; Wajner and Goodman 2011; Manoli et al. 2013; Baruteau et al. 2014; Wilnai et al. 2014; Zsengeller et al. 2014; Gallego-Villar et al. 2016; Erlich-Hadad et al. 2017). Occurrence of mental retardation is most likely multifactorial, for example cerebral damage during decompensations with metabolic encephalopathy (Kölker et al. 2006a, cerebral accumulation of toxic metabolites (Kölker et al. 2006b; Harting et al. 2008) and presumably mitochondrial dysfunction (Melo et al. 2011; Wajner and Goodman 2011) .

The demonstration of mitochondrial dysfunction is essential to improve the understanding of long-term complications and could be of major importance to develop and potentially evaluate (preventive) treatment strategies by identifying risk groups. The most reliable available evaluation of mitochondrial dysfunction is histological and biochemical assessment of a muscle biopsy. However, muscle biopsies are considered invasive and not acceptable for follow up of mitochondrial dysfunction over the years. Current plasma biomarkers, such as creatine kinase, lactate, pyruvate, alanine and proline levels, and the lactate to pyruvate ratio, have a low sensitivity and specificity (Mitochondrial Medicine Society's Committee on Diagnosis, Haas RH, Parikh et al. 2008; Davis et al. 2013, 2016). Serum fibroblast growth factor 21 (FGF-21) has been suggested as a biomarker for mitochondrial disease. Serum FGF-21 is stable with respect to processing and storage, has a higher specificity and sensitivity compared to other biomarkers, and is fast and inexpensive to determine (Suomalainen et al. 2011; Davis et al. 2013; Suomalainen 2013; Kim and Lee 2014; Ji et al. 2015; Lee 2015; Lehtonen et al. 2016; Montero et al. 2016). We studied the potential of serum FGF-21 measured in the stable metabolic period as a biomarker for long-term complications, attributed to mitochondrial failure, in patients with organic acidemias.

## Methods

### Study design

A total of 17 patients (adults and children) attending the outpatient clinic of the metabolic center of the Erasmus University Medical Center, were selected. Thereafter FGF-21 measurements were done in leftover plasma samples previously collected and stored in the laboratory biobank for research purposes. Of the 17 patients included: 11 were known with PA (cases 1–11), four with MMA (cases 12–15), and two with IVA (cases 16 and 17). The clinical courses and other laboratory findings were obtained from the medical files. The study was assigned as non-Medical Research Involving Human Subjects Act (WMO) by the Medical Ethical Committee (METC) of the Erasmus University Medical Center. Patients and/or parents of the patients gave permission for publication of their anonymous clinical and laboratory data. Patients and/or parents were informed and aware of the use of leftover material for research to which none objected. Cardiomyopathy, prolonged QT interval, renal failure, and optic neuropathy were defined as long-term complications. Severe mental retardation and growth failure were not defined as long-term complications. A severe decompensation was defined as a blood pH level below 7.1 and/or ammonia levels above 400 μmol/L (Baumgartner et al. 2014). Total protein intake was defined as natural plus synthetic protein intake.

### Data and statistical analysis

For diagnostic purposes, propionyl-CoA carboxylase activities were measured in fibroblasts at the Section Genetic Metabolic Diseases of the Erasmus University Medical Center, Rotterdam, The Netherlands. The propionyl-CoA carboxylase activity measurement is based upon the incorporation of ^14^C-labeled CO_2_ (given in the form of ^14^C-labeled sodium carbonate) within the substrate propionyl-CoA. The excess CO_2_ that was not incorporated was removed by acidification. At the same time, the incorporation of CO_2_ was also measured with pyruvate and with methylcrotonoyl as the substrate. The isovaleryl-CoA dehydrogenase activities were measured in lymphocytes based upon the anaerobic electron transfer flavoprotein fluorescence reduction assay at the Laboratory for Genetic Metabolic Diseases, Academic Medical Center, Amsterdam, The Netherlands. Serum levels of FGF-21 were determined in duplicate using a commercial ELISA kit (Millipore), which is also applied in our diagnostic process. To establish reference ranges, two hundred control subjects were studied, covering all ages, including newborns. Positive controls were 50 patients with a mitochondrial disorder known with an increased plasma FGF-21 level. We established a reference range of 0–200 pg/ml for healthy subjects, which coincides with that of Suomalainen et al. (2011). The samples were measured in duplicate and no significant differences between samples (inter-sample variability) were found. Plasma FGF-21 levels up to 3000 pg/ml were linear, and measurements were reproducible up to 4000 pg/ml. Plasma FGF-21 levels higher than 4000 pg/ml were therefore cut off to a value of 4000 pg/ml. Total protein intake estimated at several time points was based on dietary prescription by a trained metabolic dietitian during standard care, results were compared to the WHO recommended daily allowance (RDA) (Joint FAO/WHO/UNU Expert Consultation on Protein and Amino Acid Requirements in Human Nutrition (2002: Geneva et al. 2007)). Plasma amino acid levels measured during the standard of care were obtained from the medical records to determine nutritional status, and based on reference levels expressed as low (below normal reference level), normal (within reference level) or elevated levels (above reference level). The patients with MMA and PA were selected to assess the association of plasma amino acid levels and plasma FGF-21 levels. Data were summarized by frequencies, median, and means with standard deviation (SD). Normal distribution was examined by Kolmogorov-Smirnov tests and quantile-quantile (Q-Q) plots. Student’s t-tests were performed to compare means in the case of Gaussian and Wilcoxon rank sum tests (Ws) of non normal distributions. Pearson’s correlation coefficient was used to evaluate the correlation between two Gaussian distributed continuous variables and Kendall’s tau rank correlation (r_**τ**_) in the case of non normal distributions. Paired sample t-tests were performed to compare plasma FGF-21 levels in stable metabolic periods to levels in decompensations within each patient. In analyzing positive predictive value, negative predictive value, and odds ratios on the occurrence of long-term complications, we included plasma FGF-21 measurements during stable metabolic periods prior to the development of long-term complications in MMA and PA patients. In defining an optimal cut off point value for FGF-21 we used a receiver operating characteristic (ROC) curve (defining the plasma FGF-21 level with optimal sensitivity and specificity). Since protein restriction may infer FGF-21 increases, and since especially leucine and methionine restriction are associated with FGF-21 increase (Perez-Marti et al. 2016), Kendall’s tau rank correlation was calculated to test the correlation of total protein intake and methionine or leucine concentrations with plasma FGF-21 levels for each patient individually. Furthermore, we tested the association of other nutritional parameters, namely valine, isoleucine, alanine, phenylalanine, proline, and threonine, with plasma FGF-21 levels. *P*-values smaller than 0.05 were considered to indicate statistical significances. SPSS data manager (version 24) was used for statistical analyses.

## Results

### Description of the study population

Patient characteristics of all 17 patients with organic acidemia are presented in Table 1. Four patients were diagnosed by family or selective newborn screening and one patient was diagnosed prenatally. Eight patients (six with PA and two with MMA) presented in the neonatal period. Two of these patients were initially on mechanical ventilation and one was dialyzed. A total of four patients presented in childhood. These patients presented either with complaints of vomiting, malaise, and weight loss; with gastro-esophageal reflux, with encephalopathy or coma.

**Table 1:** Patient disease characteristics

Patient	Disease	Mutation	Deduced effect	Enzyme activity	Onset type; age of onset	pH at onset	Lactate level at onset (mmol/L)	NH_3_ level at onset (μmol/L)	Lowest pH*	Highest lactate level* (mmol/L)	Highest NH_3_ level* (μmol/L)
Case 1	PA	*PCAA* gene homozygous c.1409 T > G	p.(Leu470Arg)	0.03 nmol/h/mg^1^	Neonatal; 2 weeks	7.30	1.7	395	7.21	8.5	132
Case 2	PA	*PCAA* gene homozygous c.1409 T > G	p.(Leu470Arg)	ND	Prenatal diagnosis	7.39	3.3	163	7.22	6.1	255
Case 3	PA	*PCCA* gene, compound heterozygous c.625G > C/c.923dup	p.(Ala209Pro/ p.Leu308Phefs*23)	ND	Neonatal; 3 days	6.86	1.5	309	7.24	8.3	88
Case 4	PA	ND	NA	0.4 nmol/h/mg^1^	Neonatal; 3 days	7.32	2.0	667	7.35	5.8	134
Case 5	PA	ND	NA	6.6 nmol/h/mg^1^	Childhood; 5 years	7.41	ND	36	7.28	2.1	22
Case 6	PA	ND	NA	4.8 nmol/h/mg^1^	Family screening	7.40	ND	ND	7.17	1.6	90
Case 7	PA	*PCAA* homozygous C105 + 2 T > G	Splice site	0.20 mmol/h/mg^1^	Early childhood; 7 weeks	7.27	1.9	413	7.28	1.9	167
Case 8	PA	ND	NA	0.12 mmol/h/mg^1^	Neonatal; 2 weeks	7.38	NA	42	7.30	5.6	133
Case 9	PA	PCAA heterozygous c.625G > C/c.923 dup	p.(Ala209Pro)/p.(Leu308Phefs*35	0.23 mmol/h/mg^1^	Neonatal; NA	NA	NA	NA	NA	NA	280
Case 10	PA	ND	NA	0.26 nmol/h/mg^1^	Neonatal; 10 days	7.44	1.2	167	7.15	10.8	1863
Case 11	PA	*PCCB* homozygous c.1127G > A	p.(Gly376Asp)	ND	Family screening	7.32	2.9	303	7.29	4.7	165
Case 12	MMA	*MMA Mut 0*; homozygous c.2078delG	p.(Gly693Aspfs*12)	ND	Neonatal; 3 days	ND	ND	172	7.30	3.1	48
Case 13	MMA	*MMAB* gene homozygous c.556C > T	p.(Arg186Trp)	ND	Early childhood; 6 weeks	7.38	2.3	140	7.31	5.0	66
Case 14	MMA	*MMA Mut 0*; homozygous c.655A > T	p.(Asn219Tyr)	ND	Neonatal; 1 day	7.01	ND	75	7.24	6.7	106
Case 15	MMA	MMAA Homozygous c.433C > T	p.(Arg145)	0.82 nmol/17 h/mg^2^	Early childhood; 3 months	7.39	ND	48	7.33	5.8	48
Case 16	IVA	IVA heterozygous c.1241G > A	p.(Arg414Gln)	0.07 nmol/min/mg^3^	Newborn screening	7.34	3.5	49	7.31	5.2	54
Case 17	IVA	IVA homozygous c.716C > T	p.(Thr239Ile)	0.15 nmol/min/mg^3^	Newborn screening	ND	ND	ND	ND	ND	ND

* reported after initial episode. ND, not determined. NA, not available ^1^In fibroblasts, control range, 11–55 nmol/h/mg protein; ^2^ In fibroblasts, control, 3.5 nmol/17 h/mg^2^; ^3^ In lymphocytes, control range, 0.89–2.13 nmol/min/mg protein. Reference values: pH 7.35–7.45, lactate 0.5–1.7 mmol/L, NH_3_ 0–50 μmol/L

Sixteen patients showed a mild clinical course with no severe decompensations after the initial presentation (Table 1). One patient (case 10) died in the neonatal period during the first severe decompensation due to a refractory hemodynamic shock and hyperammonemia. Despite good metabolic control, eight of the remaining 16 patients developed long-term complications (Table 2). The mean age at the first long-term complication was 8.0 years (± SD: 3.4). Five PA patients developed cardiac complications, four of them (cases 1, 2, 4, and 8) developed cardiomyopathy between 8 and 13 years of age. In one patient (case 2) this was unexpected as she was diagnosed prenatally by selective screening and she always showed good metabolic control. A rapidly progressive dilated cardiomyopathy lead to cardiac failure and death at eight years of age, four months after the diagnosis of cardiomyopathy. Case 8 had mild cardiomyopathy, but died during a severe metabolic decompensation at the age of 12 years. One patient had prolonged QT interval without signs of cardiomyopathy. Four patients developed renal failure between the age of 11 months and 12 years of age. Two patients developed optic neuropathy, both at the age of 16 years. One of them was also known with reduced left ventricular function, and the other patient was already known with renal failure.

**Table 2:** Patient long-term complications

Patient	Disease	Current age	Death	Long-term complications	Cardiac complication; age at diagnosis	Renal failure; age at diagnosis	Optic neuropathy; age at diagnosis	Severe mental retardation; age at diagnosis	Growth failure
Case 1	PA	16 years	–	+	Slight reduced cardiac left ventricular function; 13 years	–	+; 16 years	+; 9 years	+
Case 2	PA	8 years	+	+	Lethal cardiomyopathy; 8 years	–	ND	+; 4 years	–
Case 3	PA	13 years	–	+	Prolonged QT interval; 9 years	–	–	–	–
Case 4	PA	12 years	–	+	Slight cardiac left ventricular hypertrophy; 9 years	–	–	–	+
Case 5	PA	35 years	–	–	–	–	ND	–	–
Case 6	PA	32 years	–	–	–	–	ND	–	–
Case 7	PA	23 years	–	–	–	–	ND	+	–
Case 8	PA	12 years	+	+	Mild cardiomyopathy; 8 years, left ventricular dilatation; 12 years	+; 12 years	ND	+	+
Case 9	PA	15 years	–	–	–	–	–	+	–
Case 10	PA	24 days	+	–	ND	ND	ND	ND	ND
Case 11	PA	4 years	–	–	–	–	ND	+	+
Case 12	MMA	17 years	–	+	–	+; 9 years	+; 16 years	–	+
Case 13	MMA	1 year 4 months	–	+	–	+; 11 months	ND	–	–
Case 14	MMA	18 years	–	+	–	+; 7 years	–	–	+
Case 15	MMA	13 years	–	–	ND	–	ND	–	–
Case 16	IVA	7 years	–	–	ND	–	ND	–	–
Case 17	IVA	9 months	–	–	ND	ND	ND	ND	–

+, present; −, absent; ND, not determined

### Overview of FGF-21 levels and long-term complications

**Fig. 1:**
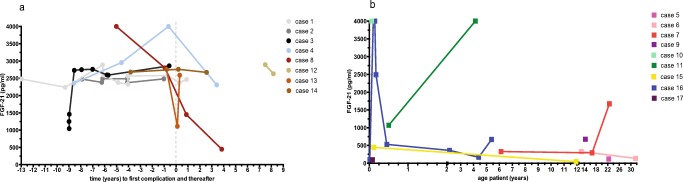
FGF-21 levels during disease progression. Dots represent patients with long-term complications (**a**) and squares those without (**b**). Line colors define the different patients. Vertical gapped line indicates the occurrence of first complication (Fig. 1a)

A total of 62 FGF-21 measurements were performed in the 17 patients. Plasma FGF-21 levels were determined before the onset of long-term complications in seven of the eight patients with long-term complications (Fig. 1a). In the remaining patient, FGF-21 measurements were performed in samples obtained after long-term complications had occurred (Fig. 1a). All FGF-21 levels from samples retrieved before long-term complications developed, were elevated (Fig. 1a). In two patients (cases 1 and 2), plasma FGF-21 levels were already elevated, up to ten times the upper level of normal, from the neonatal period onward and remained at the same level with older age. In one patient (case 3), plasma FGF-21 levels increased rapidly from the neonatal period up to eight months of age and remained high thereafter. In another patient (case 8), plasma FGF-21 levels were extremely high before complications and decreased thereafter, as previously mentioned, she eventually died of dilated cardiomyopathy. One patient (case 11) demonstrated high plasma FGF-21 levels during stable disease, at that time point without long-term complications. In an IVA patient without long-term complications (case 16), the highly elevated plasma FGF-21 levels taken during a metabolic decompensation normalized thereafter (Fig. 1b).

**Fig. 2:**
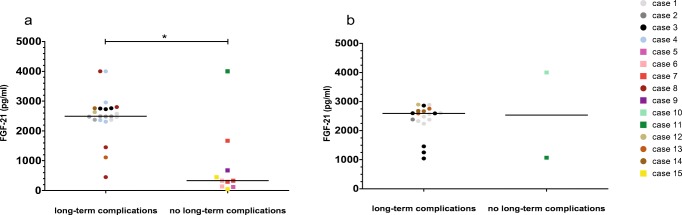
FGF-21 levels and long-term complications. Measurements during stable metabolic periods, median level indicated by horizontal line (**a**) and during decompensations, median level indicated by horizontal line (**b**). Dots represent patients with long-term complications and squares those without. Colors indicate different patients. * significant *p* value

In MMA and PA patients, median plasma FGF-21 levels (of each patient) during stable metabolic periods were significantly higher in patients with long-term complications during follow up (median FGF-21 = 2556.0 pg/ml) compared to those who did not develop complications (median FGF-21 = 287.0 pg/ml) (Ws = 29.00, Z = −2.066, *p* = 0.039) (Fig. 2a). In MMA and PA patients, the individual mean plasma FGF-21 levels during stable metabolic periods (M = 2600, SD = 843) did not differ from the individual median plasma FGF-21 levels during decompensations (M = 2309, SD = 619) (t(6) = 0.566, *p* = 0.592). None of the patients with a median plasma FGF-21 below 1500 pg/ml during the stable metabolic period (*n* = 5) developed long term complications. All, but one patient, developed long-term complications in those with a median plasma FGF-21 above 1500 pg/ml (n = 5) (samples taken during a stable metabolic period prior to the occurrence of long-term complications) in MMA and PA patients (Suppl. Fig. 1). A median plasma FGF-21 level above 1500 pg/ml during a stable metabolic period, measured before the occurrence of long-term complications, has a positive predictive value of 0.83 on long-term complications and a negative predictive value of 1.00 in MMA and PA patients.

### Renal function and FGF-21

Plasma creatinine levels did not correlate with plasma FGF-21 levels within the individual patient; nor did the median plasma FGF-21 level of each patient correlate with the median plasma creatinine level of each patient.

### Protein restriction and FGF-21

Total protein intake (g/kg/day) did not correlate with plasma FGF-21 levels in any patient, nor did median total protein intake (of each patient) correlate with median plasma FGF-21 levels (of each patient). Furthermore, total protein intake as %RDA was not associated with plasma FGF-21 levels, except in one patient (case 3) (r_**τ**_(9) = 0.957, *p* < 0.001) (Suppl. Fig. 2). In MMA and PA, plasma valine levels were low in the majority of the patients (Suppl. Fig. 3). In MMA and PA, plasma FGF-21 levels were not lower in patients with low plasma amino acids levels; valine, isoleucine, leucine, methionine, threonine, alanine, phenylalanine, and proline, respectively (Suppl. Fig. 3). Individual plasma amino acid levels were not associated with plasma FGF-21 levels, except for one patient (case 16). In this patient, all three branched chain amino acids (BCAA) were associated with plasma FGF-21 levels (valine (r(8) = −0.643, *p* = 0.026), isoleucine (r(8) = −0.618, *p* = 0.034), leucine (r(8) = −0.618, p = 0.034)).

### Age, height, and FGF-21

There is no clear association of plasma FGF-21 levels and age of the patient, in patients with long-term complications (data not shown) as well as in patients without long-term complications (Fig. 1b). In 16 of 17 patients, growth charts were available. Six out of these 16 patients had moderate to severe growth retardation (Table 2). In all of the patients, height z-score did not correlate with plasma FGF-21 levels. The median plasma FGF-21 levels did not differ significantly in patients with growth retardation (z-score < -2SD) compared to patients without growth retardation (data not shown).

## Discussion

This study demonstrates that FGF-21 can be a biomarker for long-term complications, attributed to mitochondrial dysfunction, in classic organic acidemia patients. Plasma FGF-21 levels were high in stable metabolic periods in patients with long-term complications, this was well before the onset of these complications. A median plasma FGF-21 level above 1500 pg/ml during a stable metabolic period, measured before the occurrence of long-term complications, had a positive predictive value of 0.83 on long-term complications, a negative predictive value of 1.00, and an odds ratio of 5.0. Plasma FGF-21 levels were not associated with growth, nutritional status or renal function.

Our results give additional proof to the hypothesis that mitochondrial dysfunction plays a role in long-term complications in classical organic acidemia patients (Wajner et al. 2004; Schwab et al. 2006; Chandler et al. 2009; de Keyzer et al. 2009; Fragaki et al. 2011; Melo et al. 2011; Wajner and Goodman 2011; Manoli et al. 2013; Baruteau et al. 2014; Gallego-Villar et al. 2014; Wilnai et al. 2014; Zsengeller et al. 2014; Gallego-Villar et al. 2016; Rivera-Barahona et al. 2017). Plasma FGF-21 levels were high in PA patients before they developed long-term complications, both before severe complications (such as lethal cardiomyopathy) and before milder complications (prolonged QT interval and slight left ventricular hypertrophy). Our observation of high plasma FGF-21 levels in a patient with QT prolongation, suggesting a role of mitochondrial dysfunction, is in line with Baumgartner et al. (2007). In MMA, the renal complications and the high plasma FGF-21 levels could point toward a role of mitochondrial dysfunction in renal failure, which was also proposed by others (Manoli et al. 2013). Kölker et al have suggested that mitochondrial dysfunction is not caused by a direct toxicity of methylmalonic acid but by inhibition of the respiratory chain by 2-methylcitric acid, malonic acid, and propionyl-CoA, which are increased in MMA as well (Kölker et al. 2003). Furthermore, plasma FGF-21 levels can be elevated in chronic as well as acute kidney disease and an association between plasma FGF-21 levels and estimated glomerular filtration rate has been found in non OAD patients (Hindricks et al. 2014). In our patient population we did not observe an association between plasma FGF-21 levels and renal function plasma (creatinine levels). Furthermore, plasma FGF-21 levels were already elevated before the occurrence of kidney failure, nor was there an increase in plasma FGF-21 levels when renal function deteriorated.

In stable metabolic periods there is a clear difference in plasma FGF-21 levels in patients with long-term complications compared to those without. Importantly, in this patient cohort the majority of long-term complications occurred between 7 and 9 years of age. This suggests that by determination of plasma FGF-21 levels in the stable metabolic period in early childhood we can potentially identify risk groups for long-term complications. In the stable metabolic period, only one patient (case 11) had high plasma FGF-21 levels without long-term complications. This patient had his latest visit at the age of 4 years and based on our results and the occurrence of long-term complications mainly between 7 and 9 years of age, this patient can be identified as a potential risk patient for long-term complications. During decompensation, one patient (case 16) had a severe increase in plasma FGF-21 levels, which normalized thereafter. The high plasma FGF-21 levels in this case, could be due to either the decompensation itself or the increased rigorousness of protein restriction; the last was also reflected by the decreased plasma BCAA levels observed during this decompensation. Based on our results, we recommend measuring plasma FGF-21 levels in stable metabolic periods. A median plasma FGF-21 level above 1500 pg/ml measured during a stable metabolic period, before the occurrence of long-term complications, seems to predict long-term complications. The negative predictive value of 1.00 observed in this patient cohort should be interpreted with respect to the follow-up time and the fact that long-term complications can occur at later disease stage.

A total of three patients had died, one during the neonatal period (case 10), the others at the age of 8 (case 2) and 12 years (case 8) respectively. Case 8 had initially highly elevated plasma FGF-21 levels in stable conditions, which decreased over the years until the patient died due to lethal cardiomyopathy. This decrease in plasma FGF-21 plasma levels might be the consequence of the potentially lethal mitochondrial dysfunction.

In none of the MMA and PA patients, an association between plasma FGF-21 levels and nutritional status, with respect to protein intake and plasma levels of BCAA, alanine, phenylalanine, methionine, threonine, and proline, was found.

There are some potential limitations of this study. Firstly, a retrospective study design cannot make causal inferences and therefore a prospective study is required to confirm our observations. Secondly, this retrospective design could lead to a selection bias and the relatively small number of patients studied could lead to low internal validity.

This study provides a first insight into a potential biomarker for long-term complications in organic acidemias. Long-term complications occur despite good metabolic control (Ianchulev et al. 2003; de Keyzer et al. 2009). We suggest that mitochondrial dysfunction is an ongoing process independent of metabolic control. It is important to focus on the role of preventive treatment strategies. A potential preventive therapy is anti-oxidants administration. To date, the beneficial efficacy of anti-oxidant therapies varies, ranging from improvement of long-term complications to no beneficial effect (Baruteau et al. 2014; Gallego-Villar et al. 2014; Martinez Alvarez et al. 2016; Rivera-Barahona et al. 2017). Absence of therapy efficacy can be due to the relatively late initiation of anti-oxidants in disease progression. First, the results observed in this study, namely the association between plasma FGF-21 levels and the occurrence of long-term complications as well as the high positive and negative predictive value of a median plasma FGF-21 level above 1500 pg/ml, should be replicated in another cohort of classical organic acidemia patients. Other biomarkers for mitochondrial dysfunction, like growth differentiation factor 15 (GDF-15), should be included in order to identify their role and relation with the occurrence of long-term complications in these disorders and compare them to plasma FGF-21. Once the FGF-21 results are confirmed and the role of GDF-15 established, further research should determine if FGF-21 or other mitochondrial markers could play a role in the evaluation of preventive strategies concerning the consequences of mitochondrial impairment, and if FGF-21 plays a pathogenic role in the development of mitochondrial impairment.

## Conclusion

This study provides new insights into the potential role of FGF-21 as a biomarker for predicting long-term complications in organic acidemias. Increased plasma FGF-21 levels and long-term complications are attributable to mitochondrial dysfunction. Further research should be performed in another cohort of patients to verify our observations and to investigate the potential role of FGF-21 as a biomarker of (preventive) treatment efficacy.

## Electronic supplementary material

Suppl. Fig. 1 Survival curve (percentage of nonoccurrence of long-term complications). Percentage at x-axis defines the total percentage of those without long-term complications. The occurrence of long-term complications in patients with a median FGF-21 level above 1500 pg/ml (= high), measured during a stable metabolic period before the onset of long-term complications, versus patients with a median FGF-21 below 1500 pg/ml (= low) measured during a stable metabolic period. (PNG 195 kb)

High resolution image (EPS 2041 kb)

Suppl. Fig. 2 Plasma FGF-21 levels compared to total protein intake %RDA in case 3. (PNG 88 kb)

High resolution image (EPS 1208 kb)

Suppl. Fig. 3 Plasma FGF-21 levels compared to low (below lower level of reference values), normal (within reference values) or elevated (above reference values) amino acid plasma levels. All measurements of each patient included. (PNG 457 kb)

High resolution image (EPS 4308 kb)

## Abbreviations

GDF-15: Growth differentiation factor 15
FGF-21: Fibroblast growth factor 21
IVA: Isovaleric acidemia
MMA: Methylmalonic acidemia
PA: Propionic acidemia
RDA: Recommended daily allowance
r_**τ**_: Kendall’s tau rank correlation
WHO: World Health Organization
Ws: Wilcoxon rank sum tests
