# Supplementary figures and images for: Fibroblast growth factor 21 as a biomarker for long-term complications in organic acidemias
Source: J Inherit Metab Dis. 2018 Aug 29;41(6):1179–87. doi: 10.1007/s10545-018-0244-6 (PMC6327009; doi:10.1007/s10545-018-0244-6)

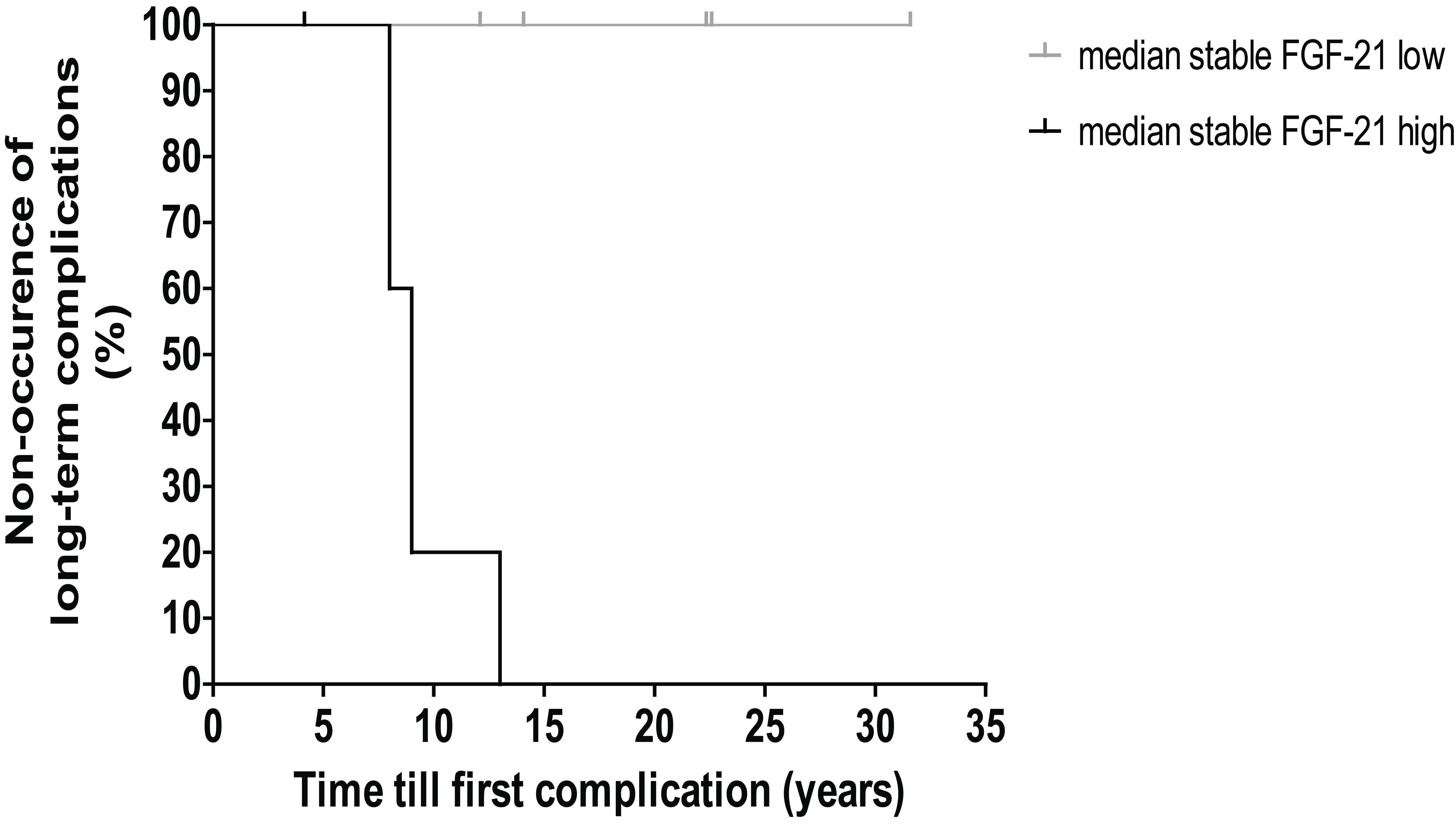

Supplement: Supplementary file 1 — Survival curve (percentage of nonoccurrence of long-term complications). Percentage at x-axis defines the total percentage of those without long-term complications. The occurrence of long-term complications in patients with a median FGF-21 level above 1500 pg/ml (= high), measured during a stable metabolic period before the onset of long-term complications, versus patients with a median FGF-21 below 1500 pg/ml (= low) measured during a stable metabolic period. (PNG 195 kb) [file 10545_2018_244_Fig3_ESM.png]

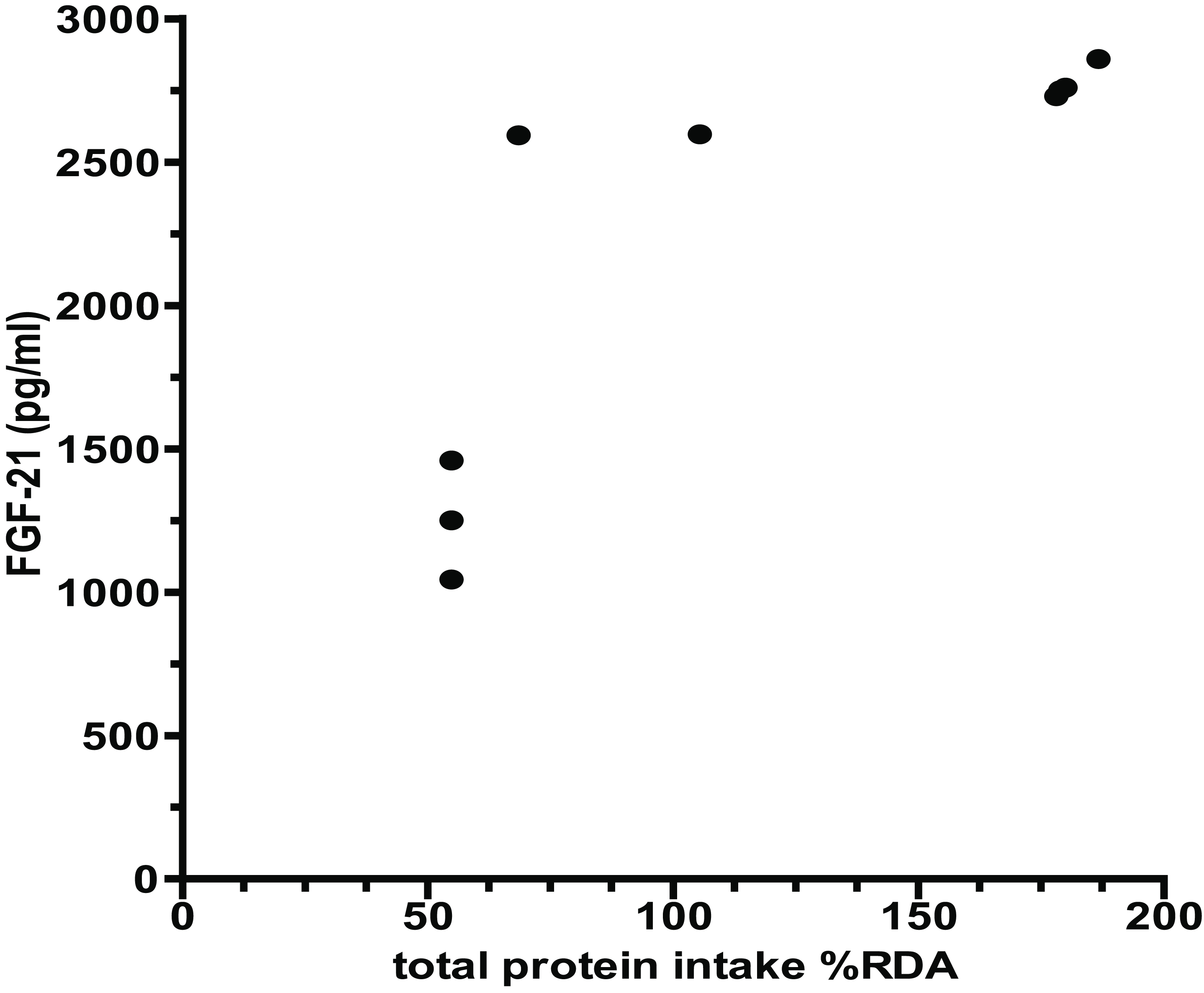

Supplement: Supplementary file 3 — Plasma FGF-21 levels compared to total protein intake %RDA in case 3. (PNG 88 kb) [file 10545_2018_244_Fig4_ESM.png]

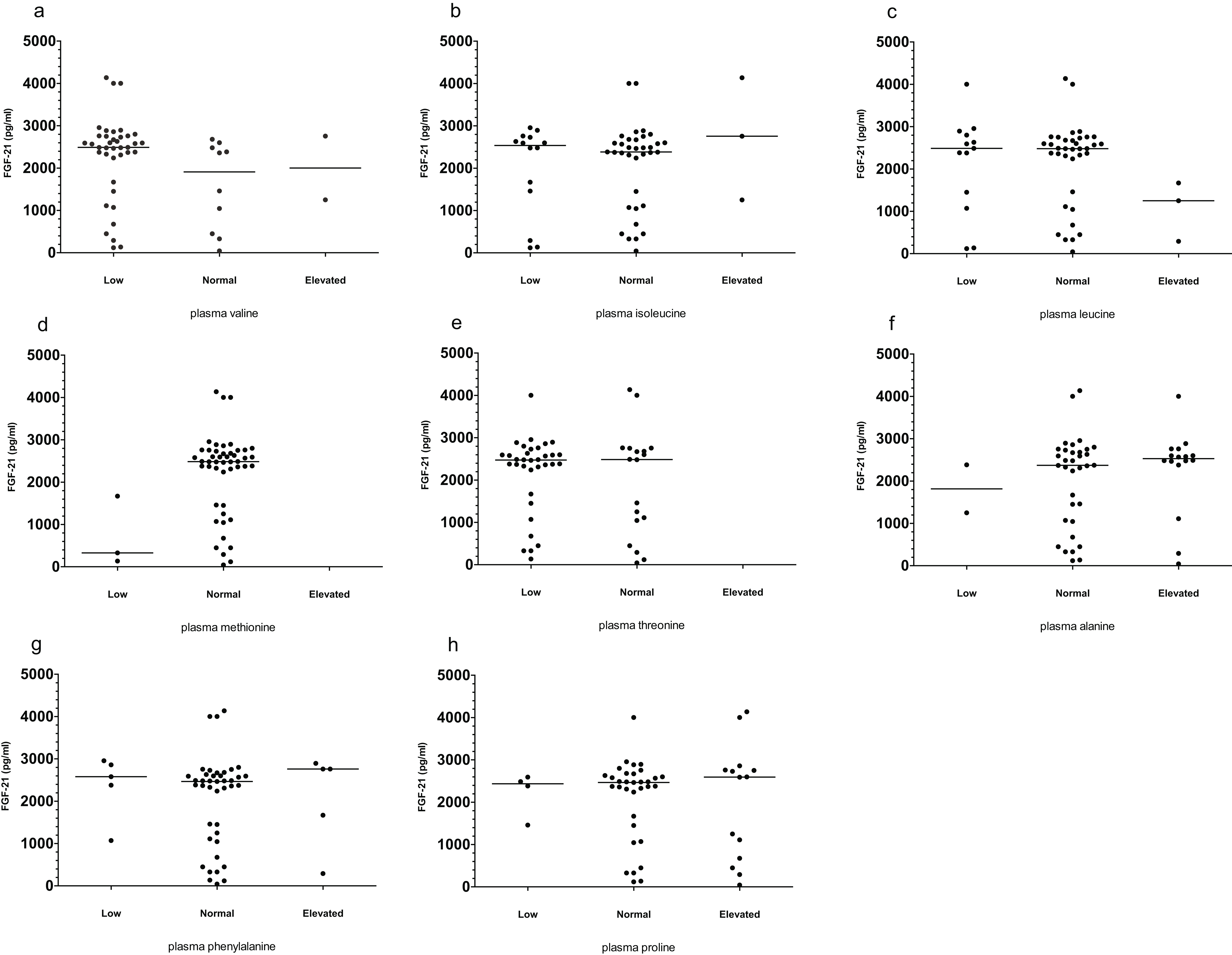

Supplement: Supplementary file 5 — Plasma FGF-21 levels compared to low (below lower level of reference values), normal (within reference values) or elevated (above reference values) amino acid plasma levels. All measurements of each patient included. (PNG 457 kb) [file 10545_2018_244_Fig5_ESM.png]
